# Development and validation of a questionnaire to evaluate the impact of COVID-19 on lifestyle-related behaviours: eating habits, activity and sleep behaviour

**DOI:** 10.1017/S1368980020004656

**Published:** 2020-11-16

**Authors:** Sakshi Chopra, Piyush Ranjan, Anita Malhotra, Anamika Sahu, SN Dwivedi, Upendra Baitha, Aastha Goel, Arvind Kumar

**Affiliations:** 1 Department of Home Science, University of Delhi, India; 2 Department of Medicine, All India Institute of Medical Sciences, New Delhi, India; 3 Department of Home Science, Lakshmibai College, University of Delhi, India; 4 Department of Psychiatry, All India Institute of Medical Sciences, New Delhi, India; 5 Department of Biostatistics, All India Institute of Medical Sciences, New Delhi, India

**Keywords:** COVID-19, Pandemic, Lifestyle-related behaviour, Questionnaire, Internal consistency, Validation

## Abstract

**Objective:**

This study was conducted to develop and validate a questionnaire to assess the impact of COVID-19 pandemic on lifestyle-related behaviour related to eating, activity and sleep pattern.

**Design:**

Indexed study used a mixed method design. Phase I employed qualitative methods for development of questionnaire including literature review, focus group discussion, expert evaluation and pre-testing. Phase II used quantitative methods for establishing construct validity of the questionnaire via parallel factor analysis.

**Participants:**

Phase 1 involved participation of experts from different fields (Departments of Medicine, Nutrition and Clinical Psychology) and general adult population. For phase II, data were collected from 124 adult respondents (female = 57·26 %); mean age (36 ± 14·8 years) residing in an urban setting.

**Results:**

The questionnaire consisted of three sections: (A) socio-demographic and anthropometric parameters, (B) twenty-four items each for investigating the changes in eating, activity and sleep behaviour before *v*. during COVID-19, (C) six items assessing COVID-19 specific reasons for lifestyle change. The Cronbach’s α value of the questionnaire is 0·83 suggesting its good internal consistency.

**Conclusions:**

This appears to be a valid tool to assess the impact of COVID-19 on lifestyle-related behaviours with potential utility for public health researchers to identify these changes at community level and develop strategies to reinforce corrective behaviours.

COVID-19 pandemic is a global burden that has far-reaching medical, social and behavioural implications. Evidence from past outbreaks has shown that as a pandemic evolves it has substantial impact on the lifestyle-related behaviours, which in turn poses a challenge in the maintenance of health and nutritional status^(1)^. The measures taken to contain the virus such as confinement and self-isolation might promote unhealthy behaviour (poor diet, sedentariness, less physical activity and disturbed sleep pattern) and distress that can potentially contribute to obesity and associated cardiometabolic risks^(2)^. It is important to understand the extent of changes in lifestyle-related behaviours and its underlying COVID-19 specific reasons to counteract these changes for maintenance of optimal health status at individual and community level. Of late, a couple of studies have used online surveys to assess the impact of COVID-19 on lifestyle-related behaviours. Although such online surveys yield data in a short period of time, they suffer the limitation of using a non-validated set of questionnaires^(3)^. Few studies have used a comprehensive list of valid questionnaires to assess significant lifestyle-related behaviour^(4,5)^. Although valid and reliable, they are more complex to administer and lack information on issues and challenges specific to the current pandemic situation.

There is a paucity of validated questionnaires that can assess the lifestyle-related changes specific to COVID-19 pandemic. We undertook this study to develop and validate an easy to administer and concise tool that will help health practitioners and researchers to understand the lifestyle changes experienced by individuals during the pandemic.

## Methodology

In this study, a mixed methods design was used for development and validation of the questionnaire. A standardised methodology was implemented in two phases: phase 1 (qualitative phase) for development of a questionnaire and phase 2 (quantitative phase) for validation of the questionnaire.

### Phase 1: Questionnaire development

**Fig. 1:**
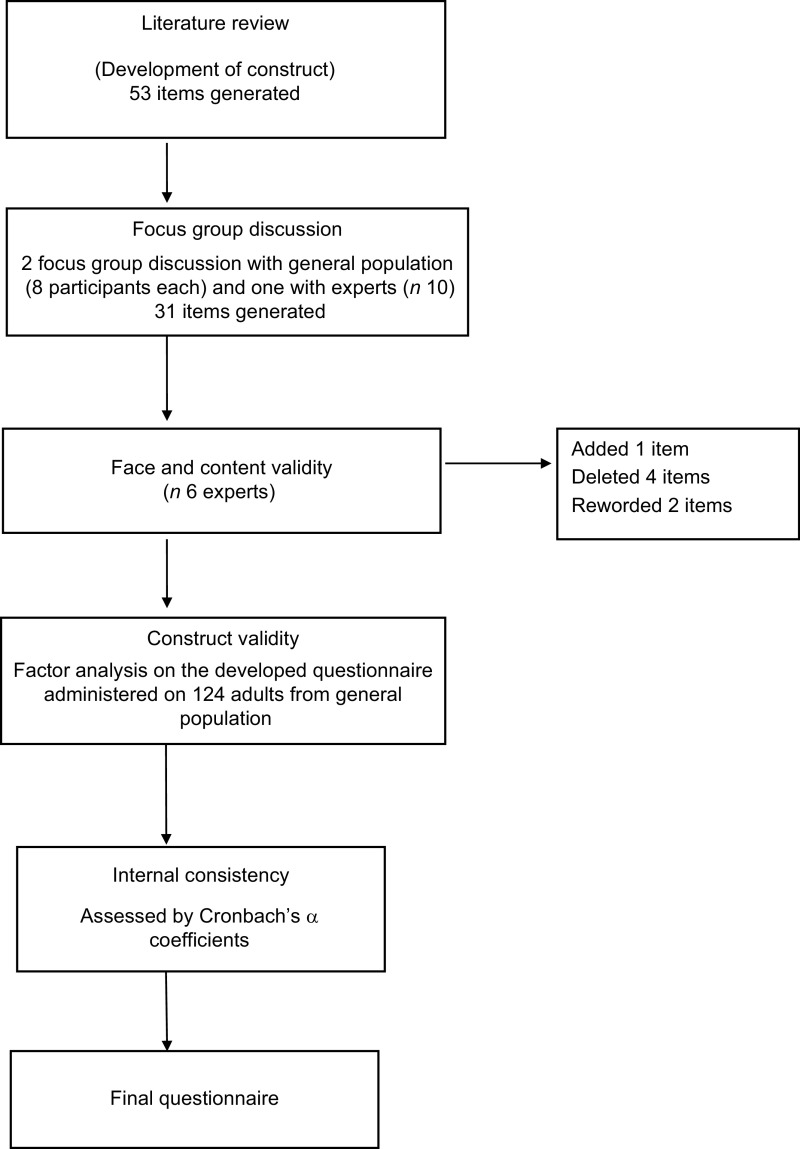
Flowchart for questionnaire development and validation

A systematic methodology was used for questionnaire development including four main steps: literature review, focus group discussion, expert evaluation and pilot testing^(6)^ (Fig. 1).

In the first step, a comprehensive literature review was carried out for the purpose of item generation on electronic search engines namely PubMed and Wiley using the given keywords string (Lifestyle* OR ‘Eating behavior’ OR Diet* OR ‘Lifestyle behaviour’ OR ‘Daily behaviour’ OR Exercise OR ‘Physical Activity’ OR Sleep*) AND (Coronavirus OR Pandemic OR COVID-19) AND (Scale OR Questionnaire OR Tool). After screening titles, abstracts and full texts, relevant papers were selected and were read in-depth to identify relevant items. The initial search resulted in a list of 561 related articles, only twenty two of which were found to be relevant. From those twenty-two articles, fifty-three items were generated.

The second step was item generation through focus group discussion (FGD). Three FGD were conducted: two with general population and a subsequent FGD with experts. The FGD with the general population included eight participants each from diverse population groups to understand the different ways in which COVID-19 had impacted their lifestyle (total participants in two FGD, *n* 16). The third FGD was with experts from different fields (physicians, gynaecologist, nutritionist, psychologist and exercise physiologist) to understand how they perceive the subject of interest to generate an exhaustive list of items (*n* 10). Eighty-four items were extracted from literature review (fifty-three items) and focus group discussion (thirty-one items). The final pool of items was categorised into three domains: eating habits, physical activity and sleep pattern. A final construct of questions was designed, ensuring no overlap. Due attention was given to ensure that the questions were framed in simple language, and worded positvely, with no ambiguity and were expressed in first person. A five-point Likert scale was used for the response options, assuming an equal distance between these options.

The generated questions were subjected to expert validation by a team of six experts from the Departments of Medicine, Nutrition, Endocrinology and Metabolism and Clinical Psychology for critical appraisal and content and face validity. Further refinement of the questionnaire was done at this stage to incorporate inputs from participants belonging to the general population after pre-testing. On the basis of their suggestions, four items were deleted (due to repetition), one item was added (related to screen time) and two items were reworded.

### Phase 2: Validity of the questionnaire

In this phase, data collection was conducted from 25 July 2020 to 28 July 2020 through a web-based questionnaire. For convenience, data were collected through Google Form (web-based questionnaire) completed by the participants or the investigator filled the forms via telephonic interview. The questionnaire was administered to 124 adult participants from diverse population groups to fulfil the principle of maximum diversity through convenience sampling method.

### Statistical analysis

Descriptive statistics were used for analysing demographics such as gender, educational status, occupation and socio-economic status. For the quantitative parameters, mean, median, standard deviation, quartile and range were calculated. Cronbach’s α was used to assess the internal consistency (i.e. the extent to which the items on the instrument measure the same thing). Cronbach’s α value of 0·7 or higher indicates good internal consistency. Parallel factor analysis was performed to examine the subdomain substructure^(7)^. This technique is used to estimate factors and/or to reduce the dimensionality of a large number of variables to a fewer number of factors. The Kaiser–Mayer–Olkin (KMO) measure is used to assess sample adequacy, and values of more than 0·5 show that the data are suitable for factor analysis. The Bartlett’s test of sphericity was used as a statistical test for the overall significance of all correlations within a correlation matrix^(8)^. Eigenvalues were calculated to represent the variance among the variables that are accounted for by a specific factor^(9)^. *P* values < 0·05 were considered as significant. The data were analysed using IBM SPSS Statistics 24 software.

## Results

The final questionnaire after the expert evaluation and pre-testing for content and face validity and establishing internal consistency has three sections (shown in Box 1) and is freely available for use. Section A comprises questions relating to general information and demographic data, self-reported anthropometric data and one question of change in weight status during COVID-19. Section B consists of two parts with twenty-four items in each. Part A (A1 to A24) assesses the baseline lifestyle-related behaviours such as eating habits, physical activity and sleep pattern, and Part B (B1 to B24) evaluates changes in different lifestyle-related behaviours during the pandemic. The domain on eating behaviour consists of twelve items on meal pattern, portion size, frequency of meals, food group consumption pattern, emotional eating and intake of high fat, salt and sugar foods and sugar-sweetened beverages consumption. The domain on physical activity pattern has six items focusing on different components of activity such as aerobic exercise, involvement in household chores, leisure-related activity, work-related sitting time and screen time. Two items were for sleep patterns, one item for daily stress levels and two items for stress-related addictive behaviours such as smoking and alcohol consumption. Section C has six items assessing the perceived COVID-19 specific reasons for changes in lifestyle-related behaviours.

### Demographic profile of the study subjects

The demographic details of the 124 participants included in validation phase are presented in Table 1. The sample had higher proportion of female respondents (57·26 %) with mean age 36 ± 14·8 years belonging to upper or upper middle economic class (77·87 %) and residing in an urban setting (94·35 %). Two-fifth of the respondents (42·7 %) reported having resumed their jobs despite the COVID-19, while almost one-fourth of them were professionals working from home (22·58 %). According to the gender, the mean BMI for women was 25·6 ± 5·2 kg/m^2^ and for men was 24·9 ± 3·6 kg/m^2^.

**Table 1:** Demographic characteristics of participants

Characteristics	Value	
Gender		
Age (years)	36·73	14·8
Male	53	42·74
Female	71	57·26
Socio-economic status		
Upper	40	32·79
Upper middle	55	45·08
Lower middle	17	13·93
Upper lower	10	8·20
Marital status		
Married	72	58·06
Single	50	48·32
Divorced	2	1·61
Family status		
Nuclear	88	70·97
Extended	17	13·71
Joint	19	15·32
Anthropometric parameters		
Self-reported height (cm)	161·1	22·6
Self-reported weight (kg)	67·7	12·7
BMI (kg/m^2^)	25·3	4·6
Women BMI (kg/m^2^)	25·6	5·2
Men BMI (kg/m^2^)	24·9	3·6

Values are presented as mean and sd or numbers and percentages.

**Figure:**
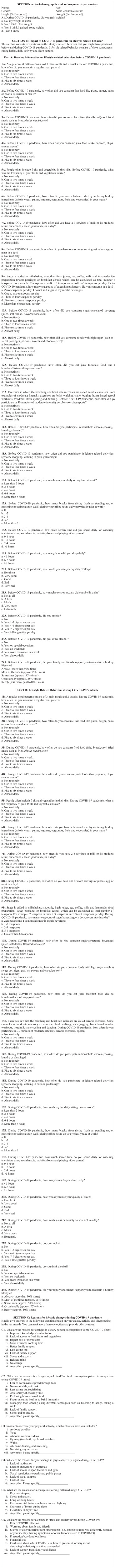

### Descriptive statistics of survey result

The response of the participants for the items listed in Sections A, B and C is given in Supplementary Table 1. Findings indicate that half of the participants (50·41%) reportedly had a stable weight followed by one-third participants (31·71%) experiencing weight gain during COVID-19. Half of the participants (50 %) maintained a regular meal pattern and added healthier ingredients to their daily meals (48·39 %). A large proportion of the participants (70 %) refrained from consuming high fat, salt and sugar foods and sugar sweetened beverages on a routine basis. It was found that only 37·10 % participants engaged in regular moderate intensity aerobic exercises (doing activity that increases breathing and heart rate) for more than 5 d per week. A majority of participants reported a little distress but maintained a regular sleep pattern with modest quality.

### Construct validity of the questionnaire

Factor analysis via Horns parallel analysis for principal components using varimax rotation was run on Section B of the questionnaire. An eigenvalue of 1 was used as a cut-off for determining the number of factors, though the scree plot also gave an estimate for the number of tenable factors. For Section B, only five factors were retained in the final questionnaire (as shown in Table 2). Overall, the total percentage of variance was 63·3 %. The questionnaire had good internal consistency, with Cronbach’s α as 0·83. In Section B, Cronbach’s α for part A and part B was 0·67 and 0·72, respectively.

**Table 2:** Eigenvalues for the factors

Component	Adjusted Eigenvalue*
1	3·03
2	2·05
3	1·22
4	1·17
5	1·04
6	0·85
7	0·81
8	0·81

* Eigenvalue > 1 were selected.

## Discussion

COVID-19 outbreak and measures of its containment has an evident impact on the lifestyle-related behaviour in the population^(10)^. Guidelines related to confinement have led to changes in daily lifestyle-related behaviours especially eating habits, physical activity and sleep pattern in a way that promotes weight gain and increases associated cardiometabolic risk. Experts believe that these lifestyle-related predictors of weight gain and cardiometabolic risk are modifiable and should be screened and addressed during COVID-19 to prevent obesity and maintain general well-being^(11)^. We have developed an easy to use and practical tool to assess the impact of COVID-19 on lifestyle-related behaviours. It is a concise and comprehensive questionnaire that enables quick assessment of important components of lifestyle-related behaviours amongst adults.

In current literature, a number of studies have assessed the changes in pre-pandemic eating, activity and sleep behaviours through rapid surveys. These surveys have broadly used two methods of assessment: (1) self-reported online survey and (2) comprehensive list of validated tools to assess different lifestyle-related behaviours^(12)^. Questionnaire-based online surveys are easy to administer and might be fairly accurate. An Italian survey to assess daily lifestyle changes during lockdown combined Mediterranean diet adherence screener with a structured questionnaire on other eating and activity behaviours such as meal frequency, snacking, sugar sweetened beverage consumption, smoking and alcohol consumption and sport^(3)^. Such assessment methods can be used for preliminary survey as these might not be truly reliable and valid to measure the changes in the lifestyle-related behaviours during COVID-19. On the other hand, a compiled list of valid and reliable scales for different behaviours such as International Physical Activity Questionnaire (IPAQ) for physical activity, Perceived Stress Scale for stress might lack specificity to current COVID-19 scenario^(13)^. Questionnaire to assess the problems faced by specific target groups such as older adults in maintenance of daily activity has also been developed and administered^(14)^. The administration of a number of valid scales might also be difficult due to high participant burden. The questionnaire developed in our study is based on significant aspects of eating, activity and sleep behaviour specific to COVID-19 with lower participant burden.

A unique feature of the developed questionnaire is that it assesses the reasons for changes in the corrective/faulty eating, activity and sleep practices. To our knowledge, there is no available questionnaire that addresses these factors related to lifestyle changes during COVID-19. Factors such as less eating out, preference of home cooked food, involvement in at-home workouts with family members and availability of time were associated with desirable eating and activity behaviours during COVID-19. Besides, the fear of coronavirus infection, lack of knowledge and motivation, lack of access to fruits and vegetables and social restrictions leading to closure of fitness centres were prime reasons for adapting unhealthy lifestyle practices. The sudden and disproportionate increase of pandemic fear amongst people followed by loss of work and financial restraints were the most probable reasons for higher reported stress and anxiety levels. Past pandemics have shown that their impact on lifestyle and mental health can last longer and have greater impact than the pandemic itself^(15)^. Moreover, it is also seen that weight gain due to positive calorie balance can be a risk factor for development of COVID-19 infections and should be assessed using different tools available in literature^(16,17)^.

The scope of useful application of this questionnaire is manifolds in the current scenario. First, the questionnaire can be used to identify the risk factors related to increasing dual burden of malnutrition and obesity during COVID-19 pandemic. Second, it can be utilised as a tool to gather lifestyle-related data while screening lifestyle-related disorders such as hypertension, diabetes, non-alcoholic fatty liver in regular clinical practices at the assessment stage^(18)^. Third, it can be used for future research to assess the impact of COVID-19 on lifestyle behaviours, results of which can drive clinical practitioners and policy makers to formulate COVID-19 specific recommendations to promote healthy lifestyle-related behaviours^(19)^.

Some limitations of this study are – inadequate representativeness from lower socio-economic strata, although efforts were made to include a diverse population; possibilities of reporting bias due to web-based survey; inability to establish predictive and concurrent validity which would require a long-term follow-up.

In conclusion, the questionnaire developed in this study provides a reliable and valid tool to assess lifestyle-related changes experienced during COVID-19 in comparison with pre-pandemic behaviours and also the probable reasons for these changes. This questionnaire has the potential to identify the specific domains of lifestyle-related behaviours that have been negatively impacted during pandemic, potentially resulting in shifting focus on implementing strategies to practice corrective behaviours. Responses from this questionnaire can also help to create awareness about the impact of COVID-19 on daily lifestyles and drive public health recommendations.

## Supplementary material

For supplementary material accompanying this paper visit https://doi.org/10.1017/S1368980020004656. click here to view supplementary material
