# Supplementary material for: Development and validation of a questionnaire to evaluate the impact of COVID-19 on lifestyle-related behaviours: eating habits, activity and sleep behaviour
Source: Public Health Nutr. 2020 Nov 16:1–16. doi: 10.1017/S1368980020004656 (PMC7783143; doi:10.1017/S1368980020004656)
Supplement: Supplementary file 1 [file S1368980020004656sup001.docx]

**Supplementary Table 1:** Frequency of participant’s responses for the validation study

| **SECTION: A** | | |
| --- | --- | --- |
| **Q.No.** | **Question** | **No. (%)** |
| A1 | During COVID-19 pandemic, did you gain weight?  No, my weight is stable | 62 (50.41) |
|  | No, I think I lost weight | 17 (13.82) |
|  | Yes, I think I gained some weight | 39 (31.71) |
|  | I don't know | 5 (4.07) |
| **SECTION:B (PART B)** | | |
| B1 | A regular meal pattern consists of 3 main meals and 2 snacks. During COVID-19 pandemic, how often do you maintain a regular meal pattern?  Not routinely | 15 (12.10) |
|  | One to two times a week | 8 (6.45) |
|  | Three to four times a week | 16 (12.90) |
|  | Five to six times a week | 23 (18.55) |
|  | Almost Daily | 62 (50) |
| B2 | During COVID-19 pandemic, how often do you consume fast food like pizza, burger, pasta or noodles as snacks or meals?  Not routinely | 93 (75) |
|  | One to two times a week | 25 (20.16) |
|  | Three to four times a week | 0 (0) |
|  | Five to six times a week | 4 (3.23) |
|  | Almost Daily | 2 (1.61) |
| B3 | During COVID-19 pandemic, how often do you consume fried food (fried bread/*poori*, fried snack such as fries, *bhujia, mathri*, etc)?  Not routinely | 70 (56.45) |
|  | One to two times a week | 36 (29.03) |
|  | Three to four times a week | 11 (8.87) |
|  | Five to six times a week | 5 (4.03) |
|  | Almost Daily | 2 (1.61) |
| B4 | During COVID-19 pandemic, how often do you consume junk foods (like popcorn, chips, candies, chocolate) as snacks?  Not routinely | 78 (62.90) |
|  | One to two times a week | 22 (17.74) |
|  | Three to four times a week | 16 (12.90) |
|  | Five to six times a week | 4 (3.23) |
|  | Almost Daily | 4 (3.23) |
| B5 | People often include fruits and vegetable in their diet. During COVID-19 pandemic, what was the frequency of your fruits and vegetables intake?  Not routinely | 9 (7.26) |
|  | One to two times a week | 10 (8.06) |
|  | Three to four times a week | 32 (25.81) |
|  | Five to six times a week | 24 (19.35) |
|  | Almost Daily | 49 (39.52) |
| B6 | During COVID-19 pandemic, how often do you have a balanced diet by including healthy ingredients (whole wheat, pulses, legumes, eggs, nut, fruits and vegetables) in your meals?  Not routinely | 6 (4.84) |
|  | One to two times a week | 8 (6.45) |
|  | Three to four times a week | 26 (20.97) |
|  | Five to six times a week | 24 (19.35) |
|  | Almost Daily | 60 (48.39) |
| B7 | During COVID-19 pandemic, how often do you have 2-3 servings of milk or its products (curd, buttermilk, cheese, *paneer* etc) in a day?  Not routinely | 12 (9.68) |
|  | One to two times a week | 9 (7.26) |
|  | Three to four times a week | 24 (19.35) |
|  | Five to six times a week | 17 (13.71) |
|  | Almost Daily | 62 (50.00) |
| B8 | During COVID-19 pandemic, how often do you have one or more servings of pulses, egg or meat in a day?  Not routinely | 7 (5.65) |
|  | One to two times a week | 14 (11.29) |
|  | Three to four times a week | 36 (29.03) |
|  | Five to six times a week | 12 (9.68) |
|  | Almost Daily | 55 (44.35) |
| B9 | Sugar is added in milkshakes, smoothie, fresh juices, tea, coffee, milk and lemonade/ food preparation (sweet porridge) or breakfast cereal; which can be calculated as total number of teaspoon: 2 teaspoon in milk + 3 teaspoons in coffee=5 teaspoons per day. During COVID-19 pandemic, how many teaspoons of sugar/honey/jaggery do you consume in a day?  Zero teaspoons per day, I don' t add sugar in my meals/ beverages | 19 (15.32) |
|  | One to two teaspoons per day | 55 (44.35) |
|  | Three to four teaspoons per day | 21 (16.94) |
|  | Five to six times teaspoons per day | 23 (18.55) |
|  | More than 6 teaspoons per day | 6 (4.84) |
| B10 | During COVID-19 pandemic, how often do you consume sugar-sweetened beverages (juice, soft drinks, flavored soda)?  Not routinely | 87 (70.16) |
|  | One to two times a week | 25 (20.16) |
|  | Three to four times a week | 8 (6.45) |
|  | Five to six times a week | 1 (0.81) |
|  | Almost Daily | 3 (2.42) |
| B11 | During COVID-19 pandemic, how often do you consume foods with high sugar such sweet porridge, pastry, sweets and chocolate etc?  Not routinely | 67 (54.03) |
|  | One to two times a week | 34 (27.42) |
|  | Three to four times a week | 17 (13.71) |
|  | Five to six times a week | 5 (4.03) |
|  | Almost Daily | 1 (0.81) |
| B12 | During COVID-19 pandemic, how often do you eat junk food/fast food due to boredom/distress/disappointment?  Not routinely | 75 (60.48) |
|  | One to two times a week | 34 (24.19) |
|  | Three to four times a week | 12 (9.68) |
|  | Five to six times a week | 4 (3.23) |
|  | Almost Daily | 3 (2.42) |
| B13 | Exercises in which the breathing and heart rate increases are called aerobic exercises. Some of the examples of moderate intensity aerobic exercises are brisk walking, static jogging, home based aerobic workouts, treadmill, static cycling and dancing. During COVID-19 pandemic, how often do you participate in 30 minutes of moderate intensity aerobic exercises/sports?  Not routinely | 48 (38.71) |
|  | One to two days a week | 12 (9.68) |
|  | Three to four days a week | 18 (14.52) |
|  | Five to six days a week | 20 (16.13) |
|  | Almost Daily | 26 (20.97) |
| B14 | During COVID-19 pandemic, how often do you participate in household chores (cooking, laundry, cleaning)?  Not routinely | 31 (25.00) |
|  | One to two days a week | 0 (0.00) |
|  | Three to four days a week | 23 (18.55) |
|  | Five to six days a week | 12 (9.68) |
|  | Almost Daily | 58 (46.77) |
| B15 | During COVID-19 pandemic, how often do you participate in leisure related activities (grocery shopping, walking in park and gardening)?  Not routinely | 90 (72.58) |
|  | One to two days a week | 0 (0.00) |
|  | Three to four days a week | 21 (16.94) |
|  | Five to six days a week | 2 (1.61) |
|  | Almost Daily | 11 (8.87) |
| B16 | During COVID-19 pandemic, how much is your daily sitting time at work?  Less than 2 hours | 26 (20.97) |
|  | 2-4 hours | 15 (12.10) |
|  | 4-6 hours-3 | 24 (19.35) |
|  | 6-8 hours | 32 (25.81) |
|  | More than 8 hours | 27 (21.77) |
| B17 | During COVID-19 pandemic, how many breaks from sitting (such as standing up, or stretching or taking a short walk) during your office hours do you typically take at work?  0 breaks | 14 (10.48) |
|  | 1-2 breaks | 31 (25.00) |
|  | 3-4 breaks | 35 (28.23 |
|  | 5-6 breaks | 28 (22.58) |
|  | More than 6 breaks | 16 (12.90) |
| B18 | During COVID-19 pandemic, how much screen time do you spend daily for watching television, using social media, mobile phone and playing video games?  0-1 hour | 12 ( 9.68) |
|  | 1-2 | 30 (24.19) |
|  | 3-4 hours | 42 (33.87) |
|  | 4-5 hours | 40 (32.26) |
|  | >5 hours | 0 (0.00) |
| B19 | During COVID-19 pandemic, how many hours do you sleep daily?  <6 hours-1 | 16 (12.90) |
|  | 6-8 hours-2 | 75 (60.48) |
|  | >8 hours-3 | 33 (26.61) |
| B20 | During COVID-19 pandemic, how would you rate your quality of sleep?  Excellent | 2 (1.61) |
|  | Very good | 15 (12.10) |
|  | Good | 52 (41.94) |
|  | Bad | 42 (33.87) |
|  | Very bad | 13 (10.48) |
| B21 | During COVID-19 pandemic, how much stressed or anxious do you feel in a day?  Not at all | 22 (17.74) |
|  | A little | 56 (45.16) |
|  | Much | 32 (25.81) |
|  | Very much | 12 (9.68) |
|  | Extremely | 2 (1.61) |
| B22 | During COVID-19 pandemic, do you smoke?  No | 2 (1.61) |
|  | Yes, 1-3 cigarettes per day | 1 (0.81) |
|  | Yes, 4-6 cigarettes per day | 0 (0.00) |
|  | Yes, 7-9 cigarettes per day | 6 (4.84) |
|  | Yes, >10 cigarettes per day | 115 (92.74) |
| B23 | During COVID-19 pandemic, do you drink alcohol?  No | 0 (0.00) |
|  | Yes, on special occasions | 1 (0.81) |
|  | Yes, on weekends | 3 (2.42) |
|  | Yes, more than once in a week | 16 (12.90) |
|  | Yes, almost daily | 104 (83.87) |
| B24 | During COVID-19 pandemic, did your family and friends support you to maintain a healthy lifestyle?  Always (more than 90% times) | 4 (3.23) |
|  | Most of the times (approx. 75% times)- | 3 (2.42) |
|  | Sometimes (approx. 50% times) | 0 (0.00) |
|  | Occasionally (approx. 25% times) | 37 (29.84) |
|  | Rarely (approx.. 10% times) | 80 (64.52) |
|  | **SECTION C**^¶^ |  |
| C1 | What are the reasons for changes in dietary pattern in comparison to pre-COVID-19 times? |  |
|  | Improved knowledge about nutrition | 62 (50) |
|  | Lack of access to fresh fruits and vegetables | 19 (15.32) |
|  | Higher cost of ingredients | 47 (37.90) |
|  | More available cooking time | 6 (4.83) |
|  | Better family support | 39 (31.45) |
|  | Less eating out  Lack of family support | 35 (28.22)  65 (52.41) |
|  | Stress and anxiety | 0 (0) |
|  | Relaxed mind | 12 (9.67) |
|  | No change | 15 (12.09) |
|  | Any other, please specify | 5 (4.03) |
| C2 | What are the reasons for changes in junk food/fast food consumption pattern in comparison to pre-COVID-19 times? |  |
|  | Fear of coronavirus spread through food | 86 (69.35) |
|  | Non-availability of cook | 50 (40.32) |
|  | Less eating out/socializing | 47 (37.90) |
|  | Availability of cooking time | 29 (23.38) |
|  | Preferring home cooked food | 58 (46.77) |
|  | Focus on eating healthy to build immunity | 52 (41.93) |
|  | Managing food craving using Different techniques such as listening to songs, taking a walk | 3 (2.41) |
|  | Lack of family support | 3 (2.41) |
|  | Stress and /or anxiety | 5 ( 4.03) |
|  | Any other, please specify | 16 (12.9) |
| C3 | In order to increase your physical activity, which activities have you included in your exercise schedule? |  |
|  | At-home aerobics | 12 (9.67 |
|  | Yoga | 48 (38.70) |
|  | At-home workout videos | 22 (17.74) |
|  | Gym (treadmill, cycle and weights) | 9 (7.25) |
|  | Walks | 51 (41.12) |
|  | At- home dancing and stretching | 14 (11.29) |
|  | Not doing any activities | 32 (25.80) |
| C4 | What are the reasons for your change in physical activity regime during COVID-19? |  |
|  | Lack of motivation | 50 (40.32) |
|  | Lack of knowledge of exercises | 39 (31.45) |
|  | Lack of access to sport facilities and gym | 21 (16.93) |
|  | Social restrictions to parks and public places | 32 (25.80) |
|  | Lack of social support | 9 (7.25) |
|  | Lack of time | 38 (30.64) |
|  | Any other, please specify____________________ | 26 (20.96) |
| C5 | What are the reasons for a change in sleeping pattern during COVID-19? |  |
|  | Daytime sleeping | 40 (32.25) |
|  | Stress and anxiety | 36 (29.03) |
|  | Long working hours | 14 (11.29) |
|  | Environmental factors such as noise and lighting | 5 (4.03) |
|  | Shortness of breath during sleep | 0 (0) |
|  | Flexibility in days’ time | 51 (41.12) |
|  | Any other,please specify_______________ | 33 (26.61) |
| C6 | What are the reasons for a change in stress and anxiety levels during COVID-19? |  |
|  | Fear of COVID-19 infection | 62 (50) |
|  | Worrying about family and friends | 66 (53.22) |
|  | Stigma or discrimination from other people (e.g., people treating you differently because of your identity, having symptoms, or other factors related to COVID-19) | 8 (6.45) |
|  | Frustration/boredom/loneliness | 40 (32.25) |
|  | Financial loss | 24 (14.35) |
|  | Confusion about what COVID-19 is, how to prevent it, or why social distancing/isolation/quarantines are needed | 14 (11.29) |
|  | Lack of support from family and friends | 2 (1.61) |
|  | Any other, please specify_____________________ | 24 (19.35) |

**Footnote:** ^¶^Participants could select multiple options.
